# Safety and efficacy of interventional embolization in cirrhotic patients with refractory hepatic encephalopathy associated with spontaneous portosystemic shunts

**DOI:** 10.1038/s41598-024-65690-1

**Published:** 2024-06-27

**Authors:** Qiao Ke, Jian He, Linsheng Cai, Xiaojuan Lei, Xinhui Huang, Ling Li, Jingfeng Liu, Wuhua Guo

**Affiliations:** 1 Department of Interventional Radiology, Mengchao Hepatobiliary Hospital of Fujian Medical University, No. 66, Jintang Road, Fuzhou, 350025 Fujian People’s Republic of China; 2 Department of Hepatopancreatobiliary Surgery, Clinical Oncology School of Fujian Medical University, No. 420, Fuma Road, Fuzhou, 350014 Fujian People’s Republic of China

**Keywords:** Cirrhosis, Hepatic encephalopathy, Spontaneous portosystemic shunts, Interventional embolization, Liver cirrhosis, Portal hypertension

## Abstract

This study aimed to assess the safety and efficacy of interventional embolization in cirrhotic patients with refractory hepatic encephalopathy (HE) associated with large spontaneous portosystemic shunts (SPSS). Inverse probability of treatment weighting (IPTW) was employed to minimize potential bias. A total of 123 patients were included in this study (34 in the embolization group and 89 in the control group). In the unadjusted cohort, the embolization group demonstrated significantly better liver function, a larger total area of SPSS, and a higher percentage of patients with serum ammonia levels > 60 µmol/L and the presence of hepatocellular carcinoma (HCC) (all *P* < 0.05). In the IPTW cohort, baseline characteristics were comparable between the two groups (all *P* > 0.05). Patients in the embolization group exhibited significantly longer HE-free survival compared to the control group in both the unadjusted and IPTW cohorts (both *P* < 0.05). Subsequent subgroup analyses indicated that patients with serum ammonia level > 60 μmol/L, hepatopetal flow within the portal trunk, the presence of solitary SPSS, a baseline HE grade of II, and the absence of HCC at baseline showed statistically significant benefit from embolization treatment (all *P* < 0.05). No early procedural complications were observed in the embolization group. The incidence of long-term postoperative complications was comparable to that in the control group (all *P* > 0.05). Hence, interventional embolization appears to be a safe and effective treatment modality for cirrhotic patients with refractory HE associated with large SPSS. However, the benefits of embolization were discernible only in a specific subset of patients.

## Introduction

Hepatic encephalopathy (HE) is a neuropsychiatric syndrome resulting from metabolic disorders induced by acute or chronic severe liver dysfunction or the presence of spontaneous portosystemic shunts (SPSS)^1,2^. The reported prevalence of overt HE among patients with decompensated cirrhosis ranges from 30 to 42%^3,4^. The symptoms of HE typically follow a recurrent pattern, leading to frequent hospitalization and imposing a considerable financial burden on the patient's family and society at large^5^. SPSS are abnormal communication channels connecting the portal venous system with the systemic circulation that form in response to portal hypertension^6^. Early investigations suggested that these shunts may reduce portal pressure to a certain extent^7,8^; however, recent research suggests that SPSS can decrease hepatic perfusion, exacerbate liver function deterioration, increase the incidence of HE, and hasten the progression of chronic liver failure^9–11^. Previous studies have demonstrated an association between HE recurrence and the presence of SPSS, revealing the presence of SPSS in 46–71% of patients with refractory HE^8,12^.

The primary treatment modality for HE is standard medical therapy (SMT); however, its efficacy is diminished in patients presenting with SPSS^1,13^. Consequently, SPSS, as a significant contributor to refractory HE, was suggested as a key target in managing this specific type of HE^6^. Several studies have demonstrated that interventional embolization can substantially ameliorate HE symptoms, reduce recurrence rates, and minimize the need for rehospitalization, without increasing the incidence of other cirrhotic decompensation events such as esophagogastric variceal bleeding (EGVB) and ascites^14–16^. However, these studies had limitations such as small sample sizes and/or a lack of control groups. Due to the lack of robust evidence, this procedure is not widely endorsed by global guidelines for the management of HE. Consequently, there is a compelling need to investigate the advantages of this procedure and the specific circumstances in which it is warranted.

The primary aim of this study was to assess the safety and efficacy of interventional embolization in cirrhotic patients with refractory HE associated with large SPSS. Furthermore, we aimed to identify specific patient subgroups that may benefit from this procedure or not.

## Materials and methods

### Study patients

This study adhered to the principles outlined in the 1975 Declaration of Helsinki and received approval from the Institutional Review Board of Mengchao Hepatobiliary Hospital of Fujian Medical University (Number: 2021_079_01). Additionally, written informed consent was obtained from all participating patients. Cirrhotic patients diagnosed with refractory HE and large SPSS between January 2017 and August 2022 were retrospectively reviewed. The exclusion criteria were: (1) history of interventions such as splenic artery embolization, splenectomy, hepatectomy, liver transplantation, surgical shunt ligation, or transjugular intrahepatic portosystemic shunt; (2) Child–Pugh score > 13; (3) patients with advanced or terminal-stage hepatocellular carcinoma (HCC) as determined by the Barcelona Clinic Liver Cancer staging system; (4) patients with refractory ascites and/or ongoing gastrointestinal hemorrhage; (5) psychiatric or neurological disorders; (6) malignancies other than HCC; (7) incomplete clinical or follow data. All eligible patients were divided into two groups based on the administration of interventional embolization: an embolization group and a control group.

### Data collection

Data on demographic characteristics, etiological factors, laboratory indices, baseline cirrhosis decompensated events, and imaging findings were extracted from the medical records. Baseline serum ammonia levels in the embolization group were collected prior to the initial embolization treatment, while in the control group, baseline serum ammonia levels were evaluated upon hospital admission with a confirmed diagnosis of refractory HE.

The Child–Pugh and Model for End-Stage Liver Disease (MELD) scores were calculated using the standard equations reported previously^17,18^, and the optimal cut-off values were determined using the "surv_cutpoint" function from the "survminer" R package. The cut-off value for the serum ammonia level was defined based on the upper limit of normal values at our center. The direction of portal blood flow was identified by liver ultrasound examination. The total area of SPSS was determined employing the established methodology detailed elsewhere^19^. Each episode of HE was assessed and classified according to the West Haven criteria^20^, and the most severe grading was selected to establish the baseline level before the initiation of treatment.

### Definition

Refractory HE encompasses two distinct manifestations: recurrent HE and persistent HE. Recurrent HE is characterized by repeated episodes occurring within a span of ≤ 6 months^20^. Persistent HE refers to an enduring pattern of behavioral alterations that continues to manifest and coexists with the recurrence of overt HE^20^. Overt HE was defined as HE with a grade ≥ II^20^.

SPSS, is characterized as an abnormal connection between the portal vein system and the systemic circulation. Large shunts are characterized by a maximum cross-sectional diameter ≥ 8 mm^9^. A solitary SPSS refers to the presence of a lone shunt, whereas multiple SPSS indicates the existence of ≥ 2 shunts. In the current study, the subtypes of SPSS included splenorenal, paraumbilical vein, gastrorenal, mesocaval, and intrahepatic portosystemic shunts^11^.

### Standard medical therapy and embolization procedures

All patients diagnosed with overt HE received SMT regardless of their undergoing embolization or not. The SMT modalities included elimination of potential precipitating factors and the administration of non-absorbable disaccharides and/or rifaximin^13^.

Interventional embolization techniques included percutaneous transhepatic obliteration (PTO), plug-assisted retrograde transvenous obliteration (PARTO), coil-assisted retrograde transvenous obliteration (CARTO) and splenic vein embolization (SVE)^21–24^. The principal embolic materials employed in these procedures were Amplatzer vascular plugs (Abbott Medical, MN, USA), coils (Cook Incorporated, Indiana, USA), or their combinations. In cases where shunts could not be completely occluded with the primary embolic materials, supplementary substances gelatin sponge and/or *N*-Butyl cyanoacrylate mixed with lipiodol were administered. All patients undergoing embolization were commenced on a regimen of rivaroxaban (orally, 10 mg, once daily) starting from the first postoperative day, and this regimen persisted for a period of three months. Simultaneously, either propranolol (orally, initiated at 10 mg twice daily with subsequent increments to the maximum tolerated dose) or carvedilol (orally, initiated at 6.25 mg and increased to 12.5 mg once daily after 1 week if tolerated) was initiated for lifelong maintenance, with careful dosage adjustments carried out in response to fluctuations in heart rate during the course of treatment.

Under local anesthesia, ultrasound guidance facilitates precise puncture, with the puncture pathways encompassing transhepatic, transsplenic, transjugular, transparaumbilical, and transfemoral veins. After successful establishment of puncture, venography was conducted via digital subtraction angiography (DSA) to reaffirm the specific shunt type and its alignment. Subsequently, the optimal embolization approach and material were selected based on the shunt type, anatomical location, and size of the SPSS. Of note, SVE is exclusively applicable to HE patients with splenorenal shunt^22^. Post-embolization, venography was performed again to confirm complete embolization of the shunt or the splenic vein. Additional variceal embolization was conducted routinely in cases with left gastric varicose vein. Measurements of hepatic venous pressure gradient (HVPG) or portal venous pressure gradient (PPG) were conducted before and after embolization.

### Endpoints and follow-up

The primary endpoint for this study was the HE-free survival during the follow-up period. HE of grade 2 or higher was considered a recurrence regardless of the presence or absence of an identifiable precipitating factor. Secondary endpoints were overall survival (OS), the changes in liver function, subgroup analyses stratified by various clinical factors, and a safety evaluation encompassing both early procedure-related complications, long-term complications and re-embolization rate.

Follow-up data of patients were obtained from electronic medical records and/or telephonic communication. Follow-up was conducted until the occurrence of patient mortality or until the last follow-up date (August 31, 2023). After the diagnosis of HE recurrence, patients had the option to persist with SMT or interventional embolization on a voluntary basis. Patients deemed suitable candidates for liver transplantation were allowed to opt for liver transplantation.

### Statistical analysis

Categorical variables were assessed using the Chi-square test or Fisher's exact test, while continuous variables were evaluated through unpaired or paired *t*-tests or Mann–Whitney U-test. HE-free survival and OS were calculated using the Kaplan–Meier method, and between-group difference was assessed using the log-rank test. Univariate and multivariate analyses were performed using the Cox proportional hazards regression model. Subgroup analyses were performed using the Kaplan–Meier method, and a forest plot of the subgroup analysis was generated, displaying each estimated hazard ratio (HR) and its corresponding 95% confidence interval (CI). Covariates associated with P-values of less than 0.05 in the univariate analysis were included in the multivariate analysis. Owing to the imbalanced baseline characteristics observed between the embolization and control groups, the inverse probability of treatment weighting (IPTW) methodology was adopted to minimize potential selection bias. IPTW was generated within a pseudo-data set employing propensity scores. These scores were estimated using a multivariate logistic regression model, wherein the treatment status (embolization or non-embolization) was used as the dependent variable, and other clinically relevant confounding factors, such as age, gender, etiology, albumin, total bilirubin, prothrombin time (PT), creatinine, serum ammonia level, Child–Pugh score, MELD score, portal blood flow, total area of SPSS, number of SPSS, maximum HE grade and HCC, were incorporated as covariates. All statistical analyses were conducted using Rstudio software (version 4.1.0). Two-tailed *P* values < 0.05 were considered indicative of statistical significance.

## Results

### Patient characteristics

**Figure 1:**
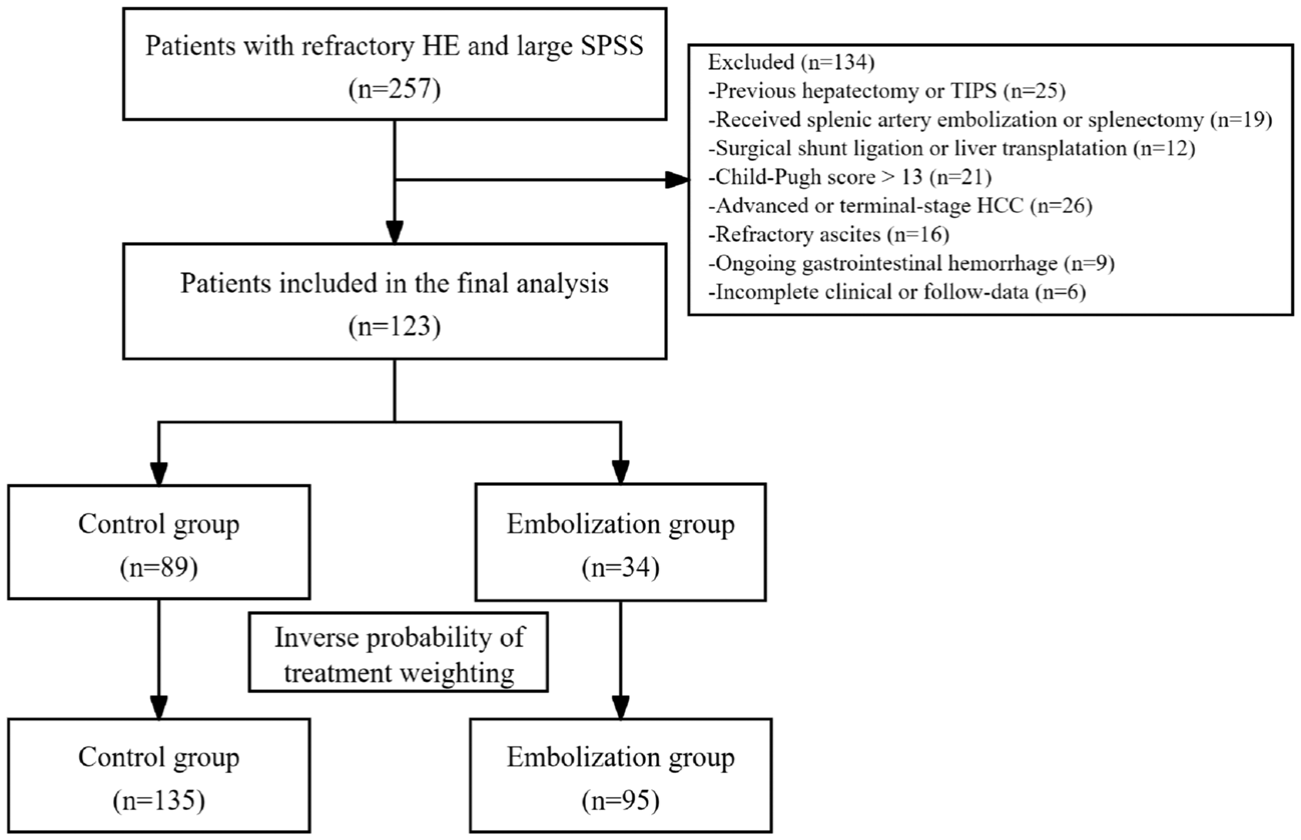
Flowchart of patient’s selection.

A total of 257 cirrhotic patients with HE and large SPSS met the eligibility criteria for this study (Fig. 1). Subsequently, 134 patients were excluded based on predetermined criteria, citing reasons such as a history of previous hepatectomy or transjugular intrahepatic portosystemic shunt (TIPS) (n = 25), prior splenic artery embolism or splenectomy (n = 19), undergoing shunt ligation or liver transplantation therapy (n = 12), having a Child–Pugh score exceeding 13 (n = 21), presenting with advanced or end-stage hepatocellular carcinoma (n = 26), experiencing refractory ascites (n = 16), enduring persistent gastrointestinal bleeding (n = 9), or possessing incomplete clinical or follow-up data (n = 6). Ultimately, a total of 123 patients were enrolled in the study, comprising 89 patients in the control group and 34 patients in the embolization group. Following IPTW, the control group consisted of 135 patients, while the embolization group comprised 95 patients.

The baseline clinical characteristics are summarized in Table 1. In the unadjusted cohort, the embolization group exhibited superior liver function, with lower total bilirubin levels, PT, Child–Pugh and MELD scores. Additionally, the embolization group had significantly larger total area of SPSS and higher percentage of patients with serum ammonia level > 60 µmol/L and presence of HCC (all *P* < 0.05). In the IPTW cohort, subsequent to the application of covariate weighting, baseline characteristics were comparable between the two groups (all *P* > 0.05).

**Table 1:** Comparison of baseline characteristics between the control and embolization groups before and after IPTW.

Variables		Unadjusted cohort			IPTW cohort		
		Control group	Embolization group	*P*-Value	Control group	Embolization group	*P*-Value
		(n = 89)	(n = 34)		(n = 135.0)	(n = 94.9)	
Age	years	56.3 ± 11.6	56.4 ± 9.5	0.976	58.7 ± 12.0	55.8 ± 8.8	0.378
Gender	Female	24 (27.0%)	3 (8.8%)	0.054	26.0 (19.2%)	11.8 (12.4%)	0.542
	Male	65 (73.0%)	31 (91.2%)		109.0 (80.8%)	83.1 (87.6%)	
Etiology	Viral	51 (57.3%)	22 (64.7%)	0.560	84.1 (62.3%)	57.2 (60.3%)	0.945
	Alcohol	24 (27.0%)	6 (17.6%)		33.0 (24.4%)	27.4 (28.9%)	
	Other	14 (15.7%)	6 (17.6%)		17.9 (13.2%)	10.3 (10.9%)	
Albumin	g/L	28.7 ± 5.8	28.8 ± 5.4	0.962	28.1 ± 5.5	29.3 ± 5.5	0.487
Total bilirubin	umol/L	62.0 [37.3–125]	36.8 [25.2–52.9]	< 0.001	84.7 [45.9–120.5]	63.2 [38.2–89.2]	0.185
PT	s	19.9 ± 5.6	17.2 ± 2.4	< 0.001	19.1 ± 4.8	18.1 ± 2.7	0.280
Creatinine	umol/L	71.0 [58.0–98.0]	75.5 [58.0–98.0]	0.248	82.0 [50.0–116.0]	78.0 [58.0–98.0]	0.552
Serum ammonia level	≤ 60 umol/L	23 (25.8%)	2 (5.9%)	0.027	47.5 (35.2%)	12.2 (12.8%)	0.187
	> 60 umol/L	66 (74.2%)	32 (94.1%)		87.5 (64.8%)	82.7 (87.2%)	
Child–Pugh score		10.2 ± 2.0	9.0 ± 2.0	0.002	9.7 ± 2.0	9.5 ± 2.2	0.685
Child–Pugh score	≤ 10	51 (57.3%)	27 (79.4%)	0.039	88.5 (65.5%)	60.6 (63.9%)	0.913
	> 10	38 (42.7%)	7 (20.6%)		46.5 (34.5%)	34.3 (36.1%)	
Meld score		16.0 [14.0–21.0]	13.0 [11.0–16.0]	< 0.001	16.0 [10.0–22.0]	15.0 [12.0–18.0]	0.359
Meld score	≤ 15	37 (41.6%)	25 (73.5%)	0.003	73.1 (54.1%)	56.1 (59.1%)	0.755
	> 15	52 (58.4%)	9 (26.5%)		61.9 (45.9%)	38.8 (40.9%)	
Portal blood flow	Hepatopetal	76 (85.4%)	25 (73.5%)	0.203	117.1 (86.8%)	73.8 (77.8%)	0.370
	Hepatofugal	13 (14.6%)	9 (26.5%)		17.9 (13.2%)	21.1 (22.2%)	
Total area of SPSS	mm^2^	150 [97–219]	249 [188–363]	< 0.001	234 [88–380]	218 [119–318]	0.739
Number of SPSS	Single	32 (36.0%)	18 (52.9%)	0.131	41.0 (30.4%)	38.9 (41.0%)	0.434
	Multiple	57 (64.0%)	16 (47.1%)		94.0 (69.6%)	56.0 (59.0%)	
HE grade, maximum	II	71 (79.8%)	30 (88.2%)	0.405	113.5 (84.1%)	86.2 (90.9%)	0.348
	III-IV	18 (20.2%)	4 (11.8%)		21.5 (15.9%)	8.7 ( 9.1%)	
HCC	No	76 (85.4%)	21 (61.8%)	0.009	92.9 (68.9%)	67.9 (71.5%)	0.868
	Yes	13 (14.6%)	13 (38.2%)		42.1 (31.1%)	27.0 (28.5%)	

IPTW, inverse probability of treatment weighting; SPSS, spontaneous portosystemic shunts; PT, prothrombin time; MELD, the model for end-stage liver disease; HCC, hepatocellular carcinoma.

The distribution of SPSS types in the two groups is presented in Table 2. In both groups, the predominant shunt types were identified as the splenorenal and paraumbilical vein.

**Table 2:** Types of SPSS in the control and embolization groups.

Type of SPSS	Control group (N = 89)	Embolization group (N = 34)
Splenorenal	22 (24.7%)	16 (47.1%)
Paraumbilical vein	23 (25.8%)	5 (14.7%)
Gastrorenal	7 (7.9%)	3 (8.8%)
Splenorenal + Paraumbilical vein	18 (20.2%)	3 (8.8%)
Gastrorenal + Paraumbilical vein	4 (4.5%)	3 (8.8%)
Others	15 (16.9%)	4 (11.8%)

SPSS, spontaneous portosystemic shunt.

### Procedural characteristics

Supplementary Fig. 1, 2 and 3 illustrates three representative embolization procedures, while Table 3 presents the procedural characteristics associated with the embolization group. All eligible patients underwent complete embolization procedures, targeting various locations including the splenorenal (38.2%), splenic vein (26.5%), paraumbilical vein (17.6%), gastrorenal (11.8%), and intrahepatic portosystemic shunts (5.9%). Puncture access was established through the transhepatic route in 15 patients (44.1%) and the transfemoral approach in 10 patients (29.4%), while the remaining nine patients underwent puncture via the transparaumbilical (14.7%) transsplenic (5.9%), or transjugular (5.9%). The embolization procedures involved the application of coils in 19 patients (55.9%), Amplatzer plugs in 11 patients (32.3%), and a combination of coils and Amplatzer plugs in the remaining four patients (11.8%). The duration of embolization was < 120 min for 16 patients (47.1%) and ≥ 120 min for 18 patients (52.9%). HVPG measurements were conducted both before and after embolization in seven patients, while PPG measurements were undertaken in five patients. The results revealed elevated values in both HVPG and PPG subsequent to embolization; however, the changes in these parameters were not statistically significant (*P* = 0.476 and *P* = 0.315, respectively).

**Table 3:** Procedural characteristics in embolization group.

Variables	Embolization group (N = 34)
Embolization location	
Splenorenal	13 (38.2%)
Splenic vein	9 (26.5%)
Paraumbilical vein	6 (17.6%)
Gastrorenal	4 (11.8%)
Intrahepatic portosystemic shunt	2 (5.9%)
Puncture pathway	
Transhepatic	15 (44.1%)
Transfemoral vein	10 (29.4%)
Transparaumbilical	5 (14.7%)
Transsplenic	2 (5.9%)
Transjugular	2 (5.9%)
Embolic materials	
Coils	19 (55.9%)
Amplatzer plugs	11 (32.3%)
Amplatzer plugs + Coils	4 (11.8%)
Embolization time	
< 120 min	16 (47.1%)
≥ 120 min	18 (52.9%)
HVPG (mmHg, N = 7)	
Preprocedural	16.3 ± 6.2
Postprocedural	19.0 ± 7.5
*P*-value for ▲HVPG	0.476
PPG (mmHg, N = 5)	
Preprocedural	15.2 ± 3.4
Postprocedural	19.4 ± 7.8
*P*-value for ▲PPG	0.315

HVPG, hepatic venous pressure gradient; ▲HVPG, the difference of HVPG before and after the embolization; PPG, portal venous pressure gradient; ▲PPG, the difference of PPG before and after the embolization.

### Long-term outcomes

**Figure 2:**
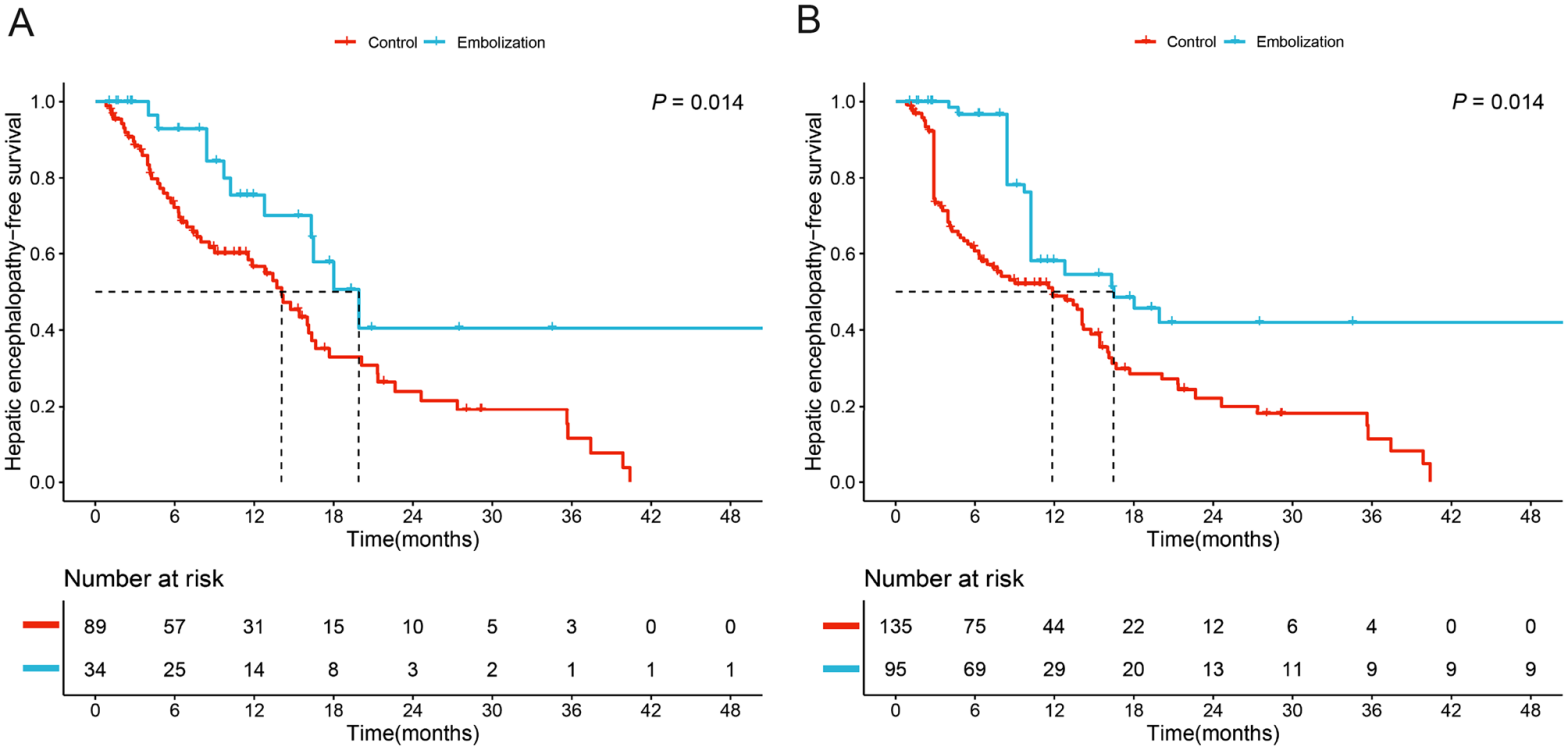
Comparison of HE-free survival between the embolization group and the control group in the unadjusted and IPTW cohorts.

In the unadjusted cohort, the recurrence rate of HE during the follow-up period was 32.4% (11/34) in the embolization group and 64.0% (57/89) in the control group. The embolization group displayed significantly longer HE-free survival compared to the control group (median HE-free survival: 19.9 months vs. 14.1 months, *P* = 0.014, Fig. 2A). A comparable outcome was noted in the IPTW cohort (median HE-free survival: 16.5 months vs. 11.8 months, *P* = 0.014, Fig. 2B).

**Figure 3:**
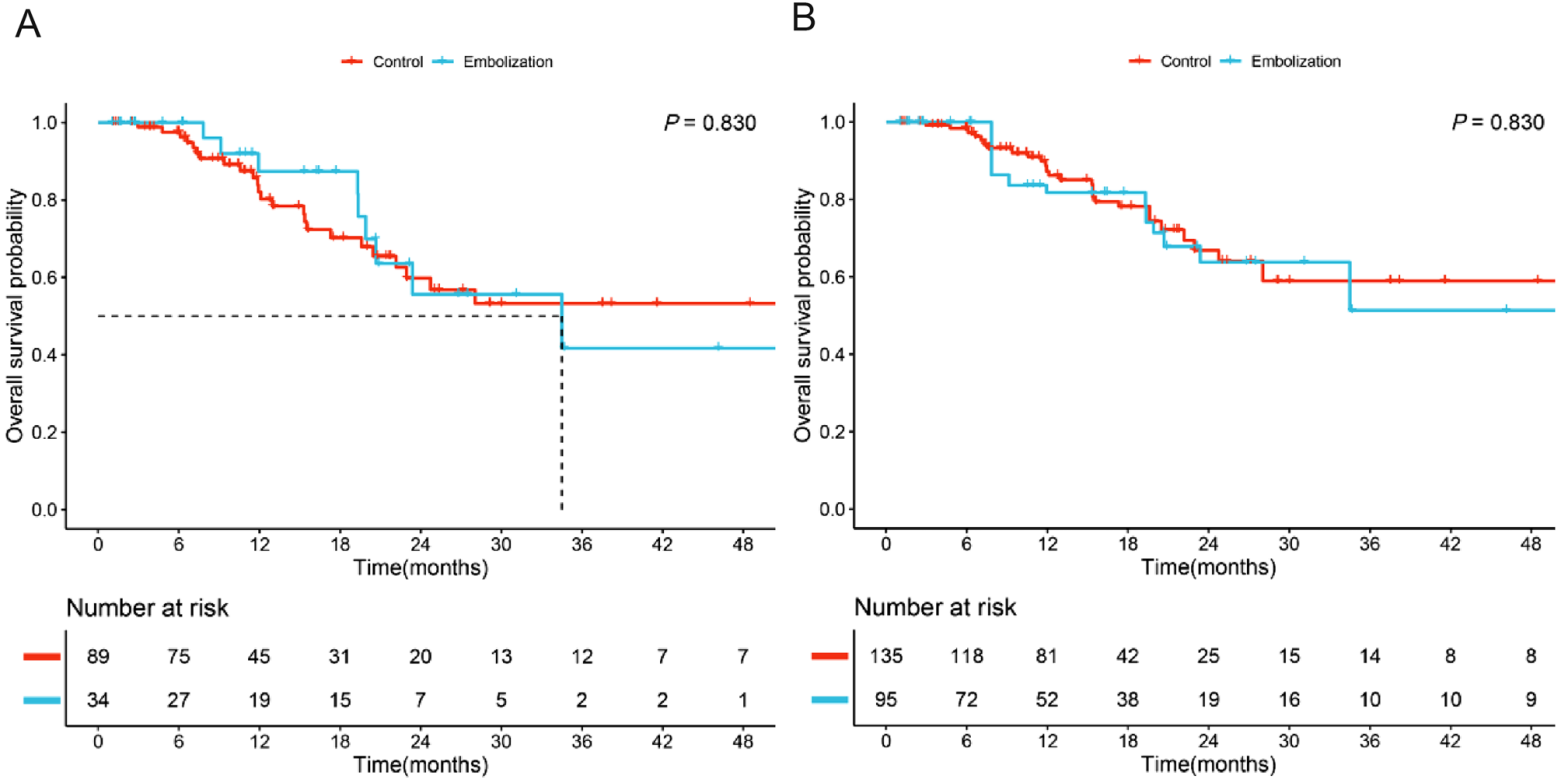
Comparison of overall survival between the embolization group and the control group in the unadjusted and IPTW cohorts.

During the follow-up period, nine patients (26.5%) died in the embolization group and 24 patients (27.0%) died in the control group, with all deaths related to liver disease. In the unadjusted cohort, Kaplan–Meier analysis showed no significant difference in OS between the embolization and control groups (median OS: 34.5 months vs. not reached, *P* = 0.830, Fig. 3A). Similar results were observed in the IPTW cohort (median OS not reached, *P* = 0.830, Fig. 3B).

The impact of embolization on liver function was assessed by changes in albumin level, total bilirubin level, PT, creatinine level, serum ammonia level, Child–Pugh scores, and MELD scores. These measurements were conducted at baseline and at the 12-month follow-up (Table 4). In the control group, the levels of albumin, PT, serum ammonia, Child–Pugh score, and MELD score exhibited a trend of deterioration at the 12-month follow-up compared to the baseline values. However, the observed differences were not statistically significant (all *P* > 0.05). In the embolization group, the levels of albumin, serum ammonia, and Child–Pugh score at the 12-month follow-up were significantly improved compared to baseline (all *P* < 0.05). At the 12-month follow-up, liver function indices in the embolization group were significantly better than those in the control group (all *P* < 0.05).

**Table 4:** Comparison of the changes in liver function parameters between the control and embolization groups.

Variables		Control group (n = 89)			Embolization group (n = 34)			*P*-value*
		Baseline	12 months	*P*-value	Baseline	12 months	*P*-value	
Albumin	g/L	28.7 ± 5.8	26.9 ± 6.0	0.053	28.8 ± 5.4	31.3 ± 4.2	0.034	< 0.001
Total bilirubin	umol/L	62.0 [37.3–125]	53.2 [35.7–127]	0.935	36.8 [25.2–52.9]	34.4 [21.8–46.9]	0.394	0.048
PT	s	19.9 ± 5.6	22.4 ± 5.3	0.219	17.2 ± 2.4	17.6 ± 5.1	0.635	0.025
Creatinine	umol/L	71.0 [58.0–98.0]	65.0 [57.0–111]	0.236	75.5 [58.0–98.0]	64.5 [53.8–80.5]	0.194	0.015
Serum ammonia level	umol/L	89.0 [60.0–119]	90.4 [66.0–141]	0.160	102.0 [82.1–153]	61.1 [51.7–89.0]	< 0.001	< 0.001
Child–Pugh score		10.2 ± 2.0	10.8 ± 2.3	0.115	9.0 ± 2.0	7.8 ± 1.9	0.018	< 0.001
Meld score		16.0 [14.0–21.0]	18.0 [14.0–24.0]	0.206	13.0 [11.0–16.0]	13.0 [10.0–16.0]	1	< 0.001

PT, prothrombin time; MELD, the model for end-stage liver disease

* for comparison between the control and embolization groups at 12 months.

### Risk factors associated with HE-free survival

The results of univariate and multivariate analyses pertaining to the HE-free survival before and after IPTW are presented in Table 5. Within the unadjusted cohort, on univariate analysis, serum ammonia level > 60 μmol/L (hazard ratio [HR] 2.50, 95% CI 1.37–4.55, *P* = 0.003), Child–Pugh score > 10 (HR 1.77, 95% CI 1.07–2.94, *P* = 0.027), MELD score > 15 (HR  1.74, 95% CI 1.07–2.84, *P* = 0.025), total area of SPSS (HR 1.01, 95% CI 1.00–1.02, *P* = 0.042), HE grade of III–IV (HR 1.76, 95% CI 1.01–3.11, *P* = 0.041), and embolization treatment (HR 0.45, 95% CI 0.24–0.86, P = 0.016) were identified as risk factors for HE-free survival. On the subsequent multivariate analysis, serum ammonia level > 60 μmol/L (HR 1.92, 95% CI 1.03–3.70, *P* = 0.039) and the administration of embolization treatment (HR = 0.66, 95% CI 0.33–0.98, *P* = 0.041) were identified as independent risk factors for HE-free survival.

**Table 5:** Univariate and multivariate analysis of hepatic encephalopathy-free survival before and after IPTW.

Characteristics		Unadjusted cohort				IPTW cohort			
		Univariate		Multivariate		Univariate		Multivariate	
		HR (95% CI)	*P-*value	HR (95% CI)	*P-*value	HR(95% CI)	*P-*value	HR (95% CI)	*P-*value
Age	years	1.01 (0.99–1.04)	0.269			1.01 (1.00–1.02)	0.041	1.01 (1.01–1.02)	0.004
Gender	Male vs Female	1.08 (0.62–1.89)	0.774			1.10 (0.59–2.10)	0.760		
Etiology	Alcohol vs Viral	1.33 (0.74–2.39)	0.334			0.80 (0.64–2.40)	0.800		
	Other vs Viral	0.92 (0.44–1.89)	0.810			0.78 (0.51–2.10)	0.760		
Serum ammonia level	> 60 vs. ≤ 60 umol/L	2.50 (1.37–4.55)	0.003	1.92 (1.03–3.70)	0.039	3.10 (1.82–5.26)	< 0.001	3.23 (1.85–5.56)	< 0.001
Child–Pugh score	> 10 vs. ≤ 10	1.77 (1.07–2.94)	0.027	1.04 (0.57–1.90)	0.907	1.60 (0.86–3.00)	0.140		
Meld score	> 15 vs. ≤ 15	1.74 (1.07–2.84)	0.025	1.50 (0.86–2.62)	0.158	1.50 (0.83–2.60)	0.190		
Portal blood flow	Hepatofugal vs. Hepatopetal	1.51 (0.84–2.71)	0.167			1.40 (0.84–2.40)	0.200		
Total area of SPSS	mm^2^	1.01 (1.00–1.02)	0.042	1.00 (0.99–1.01)	0.195	1.01 (0.99–1.02)	0.270		
Number of SPSS	Multiple vs. Single	1.39 (0.85–2.29)	0.189			1.10 (0.95–1.30)	0.240		
HE grade, maximum	III-IV vs. II	1.76 (1.01–3.11)	0.041	1.42 (0.78–2.57)	0.248	1.80 (0.99–3.30)	0.056		
HCC	Yes vs No	1.10 (0.59–2.04)	0.765			1.15 (0.53–2.56)	0.730		
Embolization treatment	Yes vs No	0.45 (0.24–0.86)	0.016	0.66 (0.33–0.98)	0.041	0.47 (0.18–0.88)	0.019	0.48 (0.20–0.96)	0.038

IPTW, inverse probability of treatment weighting; SPSS, spontaneous portosystemic shunts; MELD, the model for end-stage liver disease; HCC, hepatocellular carcinoma.

In the IPTW cohort, age (HR = 1.01, 95% CI 1.00–1.02, *P* = 0.041), serum ammonia level > 60 μmol/L (HR = 3.10, 95% CI 1.82–5.26, *P* < 0.001), and the administration of embolization treatment (HR = 0.47, 95% CI 0.18–0.88, *P* = 0.019) were identified as risk factors for HE-free survival on univariate analysis. On multivariate analysis, age (HR = 1.01, 95% CI 1.01–1.02, *P* = 0.004), serum ammonia level > 60 μmol/L (HR = 3.23, 95% CI 1.85–5.56, *P* < 0.001) and the administration of embolization treatment (HR = 0.48, 95% CI 0.20–0.96, *P* = 0.038) were identified as independent risk factors for HE-free survival.

### Subgroup analysis stratified by different characteristics

**Figure 4:**
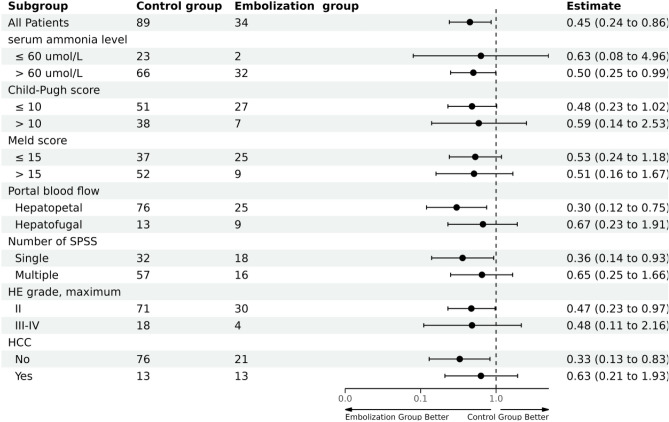
Subgroup analyses of HE-free survival stratified by different potential confounders.

We conducted subgroup analysis of HE-free survival within the unadjusted cohort, stratified by different characteristics (Fig. 4). The analysis revealed that patients with serum ammonia level > 60 μmol/L, hepatopetal flow within the portal trunk, presence of a solitary SPSS, a baseline HE grade of II, and the absence of HCC at baseline exhibited greater potential benefits from embolization treatment (all *P* < 0.05).

### Safety of interventional embolization

The safety evaluation of interventional embolization was depicted in detail in Table 6. In the embolization group, no early procedure-related complications, such as bleeding via the puncture tract, thromboembolic events, infections, or anaphylaxis, were observed. Throughout the follow-up period, the incidence of long-term complications, including EGVB (EVB: 2.9% vs. 5.6%, GVB: 0.0% vs. 7.9%, *P* = 0.140), portal vein thrombosis (17.6% vs. 9.0%, *P* = 0.301), and ascites (mild: 14.7% vs. 12.4%, moderate-severe: 5.9% vs. 20.2%, *P* = 0.156), did not significantly differ between the two groups. Additionally, two patients (5.9%) underwent secondary embolization due to the detection of recanalization of the original embolic shunt during postoperative follow-up imaging.

**Table 6:** Safety of interventional embolization.

Complications		Embolization group (N = 34)	Control group (N = 89)	*P*-Value
Early procedure-related complications				
Bleeding via the puncture tract		0 (0.0%)	–	
Thromboembolic events		0 (0.0%)	–	
Infections		0 (0.0%)	–	
Anaphylaxis		0 (0.0%)	–	
Long-term complications				
EGVB	EVB	1 (2.9%)	5 (5.6%)	0.140
	GVB	0 (0.0%)	7 (7.9%)	
Portal vein thrombosis		6 (17.6%)	9.0 (2.9%)	0.301
Ascites	Mild	5 (14.7%)	11 (12.4%)	0.156
	Moderate-severe	2 (5.9%)	18 (20.2%)	
Re-embolization rate		2 (5.9%)	–	

EGVB, esophageal and gastric variceal bleeding; EVB, esophageal variceal bleeding; GVB, gastric variceal bleeding.

## Discussion

Large SPSS are frequently identified on imaging examinations in cirrhotic patients with refractory HE^25^. Nonetheless, there is a paucity of studies exploring the safety and effectiveness of interventional embolization in this patient population^26^. In the present study, we observed that cirrhotic patients with refractory HE associated with large SPSS who underwent embolization had a prolonged HE-free survival compared to the control group. Moreover, this therapeutic approach led to a notable improvement in liver function. Additionally, administration of embolization therapy was identified as an independent risk factor of HE-free survival. Crucially, no early procedure-related complications were observed in the embolization group, and the incidence of belated complications was comparable to that in the control group.

The recently updated guidelines from the European Association for the Study of the Liver pertaining to the management of HE advocate for the embolization of SPSS in cirrhotic patients with recurrent or persistent HE, provided their MELD score is < 11^1^. However, the level of evidence supporting this recommendation is comparatively low (LoE 4, weak recommendation). Conversely, the corresponding guidelines from France do not universally advocate SPSS embolization as a standard therapeutic approach for refractory HE^27^. In a randomized controlled trial, embolization of SPSS in patients with refractory HE was found to improve the volume and synthetic functions of the liver^23^. However, longitudinal evaluation of HE recurrence in patients subjected to this intervention remains poorly evaluated. Against this backdrop, the present study undertook a cohort analysis, using IPTW to reduce potential selection bias. The results revealed a discernible reduction in the recurrence rate of HE after embolization, accompanied by significant improvements in liver function parameters. However, our findings revealed that embolization failed to confer a survival advantage in both the unadjusted and IPTW cohorts, consistent with previously documented outcomes^15^. This lack of discernible benefit may be attributed to the multivariate nature of mortality in cirrhosis. Moreover, the retrospective nature of the current studies, coupled with limited sample sizes and short follow-up durations, hampers the ability to adequately assess long-term efficacy. Therefore, large-sample randomized controlled trials are warranted to further validate the effect of embolization on OS.

Contemporary studies have presented a broad spectrum of embolization modalities including PTO, balloon-occluded retrograde transvenous obliteration (BRTO), as well as CARTO and PARTO, which have emerged as subsequent refinements to BRTO^6^. Initial accounts primarily centered on the utilization of PTO, yielding demonstrable efficacy in reducing the recurrence of HE after embolization^14^. BRTO, initially devised for the management of gastric variceal hemorrhage, has more recently been found to be useful in HE patients with splenorenal and gastrorenal shunts^28^. However, it is imperative to acknowledge the potential elevation in portal pressure subsequent to embolization, potentially exacerbating complications associated with portal hypertension, including gastroesophageal varices and ascites^6,29^. Furthermore, the use of sclerotic agents may cause adverse effects such as pulmonary edema or portal vein thrombosis^30^. Alternatives such as CARTO and PARTO have yielded comparable outcomes with a lower incidence of adverse effects^24,31^. Another strategic approach involves selective SVE, which serves as a paradigm of preserving the shunt while effecting disconnection between the portal and systemic circulations^22,32^. This approach can help reduce HE recurrence; however, its application is restricted to patients with splenorenal shunts^22,32^. Consequently, additional studies are warranted to discern the optimal interventional embolization modality.

Safety is a pivotal criterion in the evaluation of treatment options. Despite the promising outcomes of embolization, it is not widely used due to the risk of severe complications. These complications include those associated with procedural aspects and those emerging subsequent to the embolization procedure. Early procedure-related complications include bleeding, thromboembolic events, infection, and anaphylaxis^6^. Embolization can also lead to long-term complications, including the aggravation of portal hypertension, such as de novo occurrence or aggravation of preexisting esophageal and gastric varices (EGV, with or without bleeding), ascites, or the development of novel collaterals^6,29^. In previous studies, patients with severe or refractory ascites, as well as those presenting with large EGVs, were considered ineligible for the procedures^6,29^. This may have led to the low rates of complications observed following embolization procedures. The choice of embolization modality can influence the overall incidence of complications. Compared to other procedures, SVE has a relatively mild impact on portal pressure elevation, thereby contributing to a comparably lower rate of complications^22,33^. Nevertheless, there is a paucity of studies entailing a comprehensive assessment of its safety. In the present study, no early procedural complications were observed in the embolization group, and the occurrence of long-term postoperative complications was comparable to that in the control group. These findings suggest that embolization is a safe treatment for cirrhotic patients with refractory HE related to SPSS.

Notably, one size did not fit all, and the benefits of embolization were not uniformly observed across all patients. Several studies have identified preoperative MELD score as a potential prognostic indicator. Specifically, when the MELD score is > 11, embolization yields negligible benefits and can potentially cause severe complications^6,14^. In the study by An et al^15^, favorable outcomes of embolization were observed only in patients with a MELD score < 15 and absence of HCC. Furthermore, Choi et al.^34^ developed the Albumin-Bilirubin-INR scoring model, which showed good ability to predict the survival rates at the 3-month and 6-month follow-up after embolization. In this study, on subgroup analyses, patients with preoperative ammonia level ≥ 60 μmol/L, hepatopetal flow within the portal trunk, those with a solitary SPSS, baseline HE grade of II, and those without HCC at baseline were found to significantly benefit from the embolization treatment. These findings indicate that embolization therapy is applicable only to a specific subgroup of patients.

Some limitations of this study warrant acknowledgment. First, this was a single-center retrospective study with a small sample size; therefore, our results may be influenced by selection bias. However, we employed IPTW to minimize this bias. Second, the eligible patients exhibited a diverse array of spontaneous SPSS types, alongside varying embolization treatment modalities, potentially introducing a confounding influence on the outcomes. Third, the subgroup analysis was deprived from unadjusted cohort, so the result should be interpreted with caution. A larger prospective, multi-center study is warranted to validate these findings.

## Conclusion

Interventional embolization was found to be associated with prolonged HE-free survival and improved liver function in cirrhotic patients with refractory HE related to SPSS. No early procedure-related complications were observed in our cohort, and the incidence of long-term complications was comparable to that in the control group. However, this intervention seems to be effective in specific patient cohorts.

### Supplementary Information

Supplementary Figures.

## Data Availability

Data from this study are available upon request from the corresponding author WHG (Email: guowuhua@aliyun.com).
