# Supplementary material for: Safety and efficacy of interventional embolization in cirrhotic patients with refractory hepatic encephalopathy associated with spontaneous portosystemic shunts
Source: Sci Rep. 2024 Jun 27;14:14848. doi: 10.1038/s41598-024-65690-1 (PMC11211343; doi:10.1038/s41598-024-65690-1)

**Supplementary Fig 1.** Representative images of splenorenal shunt and percutaneous transhepatic obliteration (white arrow shows the splenorenal shunt; A, axial contrast-enhanced CT scan in portal venous phase; B, coronal contrast-enhanced CT scan in portal venous phase; C, superior mesenteric venography before embolization; D, superior mesenteric venography after embolization).


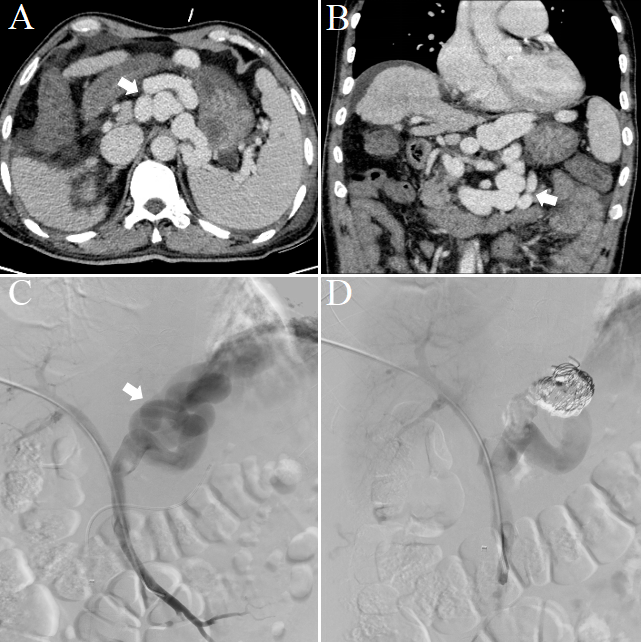


**Supplementary Fig 2.** Representative images of gastrorenal shunt and plug-assisted retrograde transvenous obliteration (white arrow shows the gastrorenal shunt; A, axial contrast-enhanced CT scan in portal venous phase; B, coronal contrast-enhanced CT scan in portal venous phase; C, shunt venography before embolization; D, X-ray radiography after embolization).


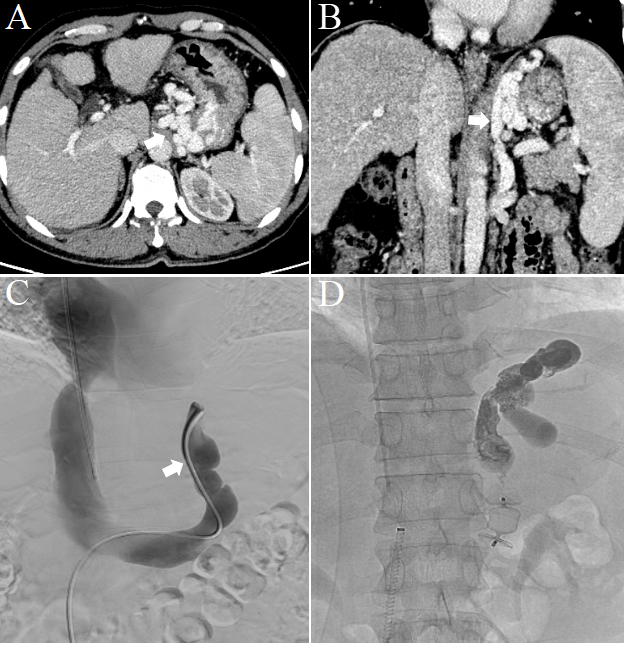


**Supplementary Fig 3.** Representative images of splenorenal shunt and splenic vein embolization (white arrow shows the splenorenal shunt; A, axial contrast-enhanced CT scan in portal venous phase; B, coronal contrast-enhanced CT scan in portal venous phase; C, superior mesenteric venography before embolization; D, superior mesenteric venography after embolization).


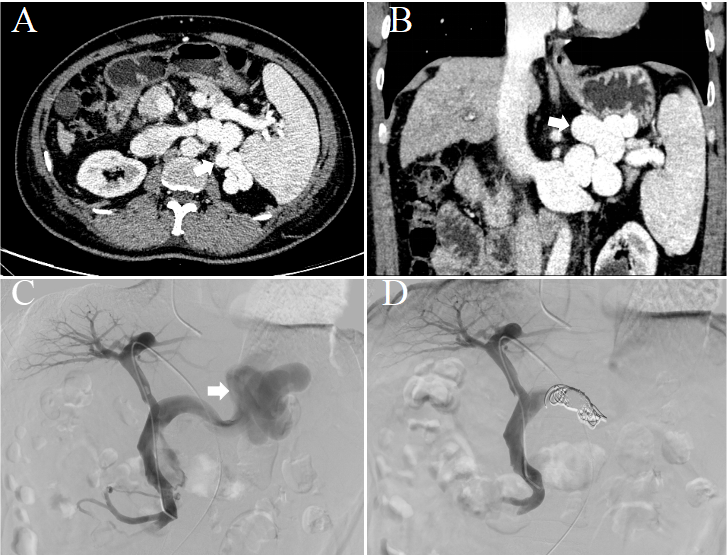

Supplement: Supplementary file 1 — Supplementary Figures. [file 41598_2024_65690_MOESM1_ESM.docx]
